# Draft genomic sequences of a rare environmental pathogen, *Comamonas kerstersii*, from immunocompromised patients with acute gastroenteritis

**DOI:** 10.1128/mra.00998-24

**Published:** 2025-04-30

**Authors:** Mamatha Ballal, Sohan Rodney Bangera, Vignesh Shetty, Shashikiran Umakanth, Lauge Holm Sørensen, Rene S. Hendriksen

**Affiliations:** 1 Enteric Diseases Division, Department of Microbiology, Kasturba Medical College, Manipal Academy of Higher Education 76793 , Manipal, India; 2 Parasites and Microbes Programme, Wellcome Sanger Institute, Hinxton, Cambridge, United Kingdom; 3 Department of General Medicine, Dr TMA Pai Hospital 75186 , Udupi, India; 4 Research Group for Global Capacity Building, National Food Institute, Technical University of Denmark, Kongens Lyngby, Denmark; 5 Bioinfomatics, Rigshospitalet, Copenhagen, Denmark; Indiana University Bloomington, Bloomington, Indiana, USA

**Keywords:** Comamonas kerstersii, rare environmental pathogen, acute gastroenteritis, immunocompromised hosts

## Abstract

*Comamonas kerstersii* is a ubiquitous, aerobic, motile gram-negative bacteria considered commensal but has played a significant role as a potential pathogen causing clinical infections. Here, we report the genomic sequences of five *C. kerstersii* isolated from immunocompromised patients with acute gastroenteritis.

## ANNOUNCEMENT

*Comamonas kerstersii*, a gram-negative bacterium of the Comamonadaceae family, is globally found in water, soil, and gut microbiota (1). It is linked to abdominal and gastrointestinal infections, often in polymicrobial environments. Its role in diarrhea is unclear, typically associated with gastrointestinal perforations (2). We report draft genomic sequences of five C. *kerstersii* strains from immunocompromised patients with acute gastroenteritis. The study was approved by the Kasturba Medical College and Hospital Ethics Committee under approval number IEC1: 180/2024.

The strains were isolated in 2014 from patients with diarrhea admitted to a tertiary care hospital in India. Stool specimens were collected for routine microbiological investigation. The isolates were non-lactose-fermenting on MacConkey agar. Gram staining identified them as gram-negative bacilli. The isolates were motile by the hanging drop method, catalase positive, oxidase positive, indole negative, urea not hydrolyzed, citrate not utilized, and showed both slant and butt as alkaline on TSI medium without H₂S production. No hemolysis was observed on blood agar. These biochemical reactions may not conclusively identify the bacteria as Comamonas. For definitive identification, MALDI-TOF mass spectrometry confirmed the identification as Comamonas. The strains were subcultured on nutrient agar slopes, stored at 4°C, and then inoculated into brain heart infusion broth for 18–24 hours. Genomic DNA was extracted from the broth using the Invitrogen Easy-DNA Kit, and DNA concentrations were determined with the Qubit dsDNA BR assay kit. Paired-end libraries were prepared using the modified NEB library prep kit and sequenced (2 × 150 bp) on Illumina Hiseq, yielding average insert sizes of 255–259 bp. Bioinformatic tools were run with default settings unless otherwise stated. Trimming was performed with AdapterRemoval v. 1.1 (https://github.com/MikkelSchubert/adapterremoval) and *de novo* assembled with VelvetOptimiser v. 2.2.5 (https://github.com/tseemann/VelvetOptimiser/tree/2.2.5) with a maximum k-mer length of 99 and Velvet v. 1.2.07 (3). Estimated completeness, contamination, and routine QC metrics were found with CheckM2 v. 1.0.2 (4). Antimicrobial resistance genes and plasmids were predicted using ResFinder v. 4.5.0 (5) and PlasmidFinder v. 2.1 (6) ().

PathogenFinder v. 1.1 (http://cge.food.dtu.dk/services/PathogenFinder) (7) indicated C3 (ERS2626704) and C4 (ERS2626705) of the *C. kerstersii* as potential pathogens. VirulenceFinder v. 1.5 (https://cge.food.dtu.dk/services/VirulenceFinder/) (8) detected celb- Endonuclease colicin E2 in isolate C3 (ERS2626704). Assemblies were searched against VFDB (21-10-2024) (9) using blastn v. 2.16+ (10) identifying diverse potential virulence genes. All isolates contained analogs for secretion systems IV, VI, and motility proteins (motA, fliAIP). C1–C4 additionally contained system III proteins and fliN.

**Fig 1:**
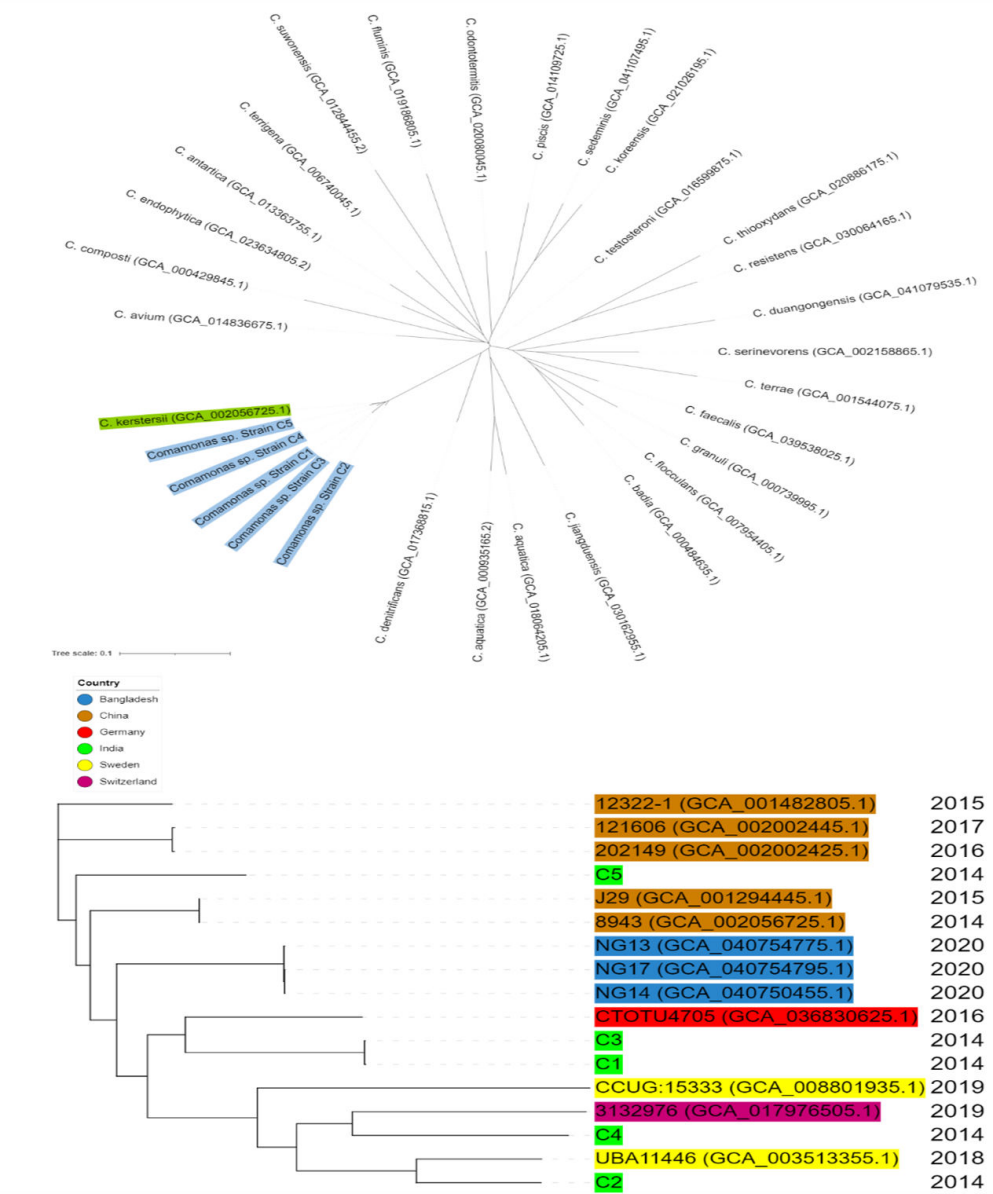
Phylogenetic tree with SNPs of five strains of *C. kerstersii,* strains C1–C5, and NCBI reference genomes for each *Comamonas* spp. Strains C1–C5 are highlighted in blue; closest reference genome [*C. kerstersii*] is highlighted in green. C1–C5 and 12 high-quality draft geno*mes of C. kerstersii*. Each strain is highlighted by country with year of isolation. NCBI genomes downloaded from the GenBank database. Visualized in Itol v. 6.9.1 [ref]. Genomes for *C. testosterone* sp. [GCA_016599875.1] and *C. kerstersii* sp. [GCA_002056725.1] were used as references for the top and bottom phylogenetic trees, respectively. Figure created with BioRender.com.

Single nucleotide polymorphism phylogenetic analysis using CSI Phylogeny v. 1.4 (https://cge.food.dtu.dk/services/CSIPhylogeny/) and FastTree v. 2.1.11 (11) produced maximum-likelihood phylogenies and a Mash v. 2.3 (12) comparison to reference genomes for *Comamonas* spp. from NCBI Genbank (genomes listed in). A phylogeny of strains C1–C5 with high-quality genomes of *C. kerstersii* from NCBI (*n* = 12) shows that C1 and C3 cluster closely together with considerable distance to and between C2, C4, and C5 (Fig. 1). Strains C1–C4 are more related to wastewater and feces isolates from the European countries. Strain C5 appears more related to clinical isolates from China. The diversity of the isolates indicates sporadic community infection.

## Data Availability

A complete list of genomic sequence data is available at https://doi.org/10.6084/m9.figshare.28191680. Reference genomes for *Comamonas* spp. from NCBI Genbank are listed at https://doi.org/10.6084/m9.figshare.28485461.v1. Raw sequence data have been submitted to the European Nucleotide Archive under study accession numbers ERS2626702, ERS2626703, ERS2626704, ERS2626705, ERS2626706.
